# Sporadic amyotrophic lateral sclerosis with seropositive neuromyelitis optica spectrum disorder: A case report

**DOI:** 10.1097/MD.0000000000025580

**Published:** 2021-04-23

**Authors:** Jin Young Kim, Hye Jeong Oh, Yuntae Kim, Jin Myoung Seok

**Affiliations:** a Department of Physical Medicine and Rehabilitation, Soonchunhyang University Hospital Bucheon, Soonchunhyang University College of Medicine, Bucheon; b Department of Neurology; c Department of Physical Medicine and Rehabilitation, Soonchunhyang University Hospital Cheonan, Soonchunhyang University College of Medicine, Cheonan, Korea.

**Keywords:** amyotrophic lateral sclerosis, anti-aquaporin-4 antibody, neuromyelitis optica spectrum disorder

## Abstract

**Rationale:**

Neuromyelitis optica spectrum disorder (NMOSD) is a severe inflammatory disorder of the central nervous system with an autoantibody against aquaporin-4 protein (AQP4), and amyotrophic lateral sclerosis (ALS) is a fatal neurodegenerative disease. We report a female patient with ALS who had asymptomatic AQP4 antibody at the diagnosis of ALS, and NMOSD occurred 4 years later after the diagnosis of ALS.

**Patient concerns:**

She was already bedridden and had tracheostomy because of ALS which was diagnosed at her age of 55. At the time of her ALS diagnosis, she had no brain or spinal cord lesions, but was seropositive for AQP4 antibody. At her age of 59, new-onset complete paralysis of all extremities and severe pain on the posterior neck and both shoulders occurred and visited the hospital.

**Diagnosis:**

Longitudinally extensive transverse myelitis was diagnosed, which was the onset attack of seropositive NMOSD. The diagnosis was confirmed based on the international consensus diagnostic criteria for NMOSD with MR imaging, cerebrospinal fluid exam and laboratory work-ups with AQP4 antibody test.

**Interventions:**

High dose methylprednisolone was administered for 5 days. Plasma exchange as a further treatment was recommended, but she and her family refused.

**Outcomes:**

Her pain was relieved after steroid treatment, but there was no improvement of her leg weakness.

**Lessons:**

This case is a rare combination of neuroinflammatory and neurodegenerative diseases. Considering the alterations of blood-brain barrier along with the progression of ALS, it highlights that the consequence of ALS pathogenesis might affect the development of NMOSD. And the careful follow-up is recommended even in patients with profound weakness, especially if those who were at risk of developing certain neurological disorders.

## 1 Introduction

Neuromyelitis optica spectrum disorder (NMOSD) is a severe inflammatory disorder of the central nervous system (CNS) with an autoantibody against aquaporin-4 protein (AQP4). AQP4 antibody was known to be pathogenic, but for the lesion formation, the antibody should gain access to the CNS across the blood-brain barrier (BBB) with cellular/humoral immune factors.^[1]^ There have been reported cases of long-term asymptomatic AQP4 antibody positive carriers, however, their initial events or mechanisms which could cause increased BBB permeability and NMOSD were not well understood.^[2,3]^ Amyotrophic lateral sclerosis (ALS) is a fatal neurodegenerative disorder characterized by loss of motor neurons. The pathomechanism of ALS is not well known, the compromised BBB integrity has been recognized as a possible factor for disease pathogenesis and progression.^[4]^ Here, we report a female patient with ALS who had asymptomatic AQP4 antibody at the diagnosis of ALS, and NMOSD occurred 6 years later after the onset of ALS.

## 2 Case report

The patient presented with weakness in left upper extremity for the first time at the age of 53. Muscle weakness progressed slowly to bilateral upper and lower extremities with increased deep tendon reflexes, but no pain or sensory symptoms. Laboratory, imaging studies, and electrodiagnosis were performed for the diagnosis of ALS. Brain and spine magnetic resonance imaging (MRI) revealed no abnormal findings. Laboratory findings were normal except for AQP4 antibody. She had AQP antibody positivity by tissue-based indirect immunofluorescence assay, which was done for the differential diagnosis. Electrodiagnosis showed widespread denervation in all limbs compatible with ALS. She was diagnosed as having ALS since the age of 55. And she became bedridden and had tracheostomy because of the progression of the disease. Her upper extremities were weak; the strength of finger flexor and extensor muscles was Medical Research Council (MRC) grade 2, and her leg muscles showed MRC grade 3. But the sensation was normal. Deep tendon reflexes showed hyperreflexia in all 4 limbs in a symmetrical fashion.

At her age of 59, she presented to the emergency room with new-onset complete paralysis of all extremities and severe pain on the posterior neck and both shoulders. Her newly developed pain and quadriplegia had started 5 days ago and had acutely progressed. On admission, her neurologic exams showed that there were profound weakness and atrophy of all extremities with MRC grade 0 except her both fingers which showed MRC grade 2. And there was a sensory level at T4 with sensory loss in the lower limbs. Extensor plantar response was present on both sides. She also had bladder dysfunction.

**Figure 1:**
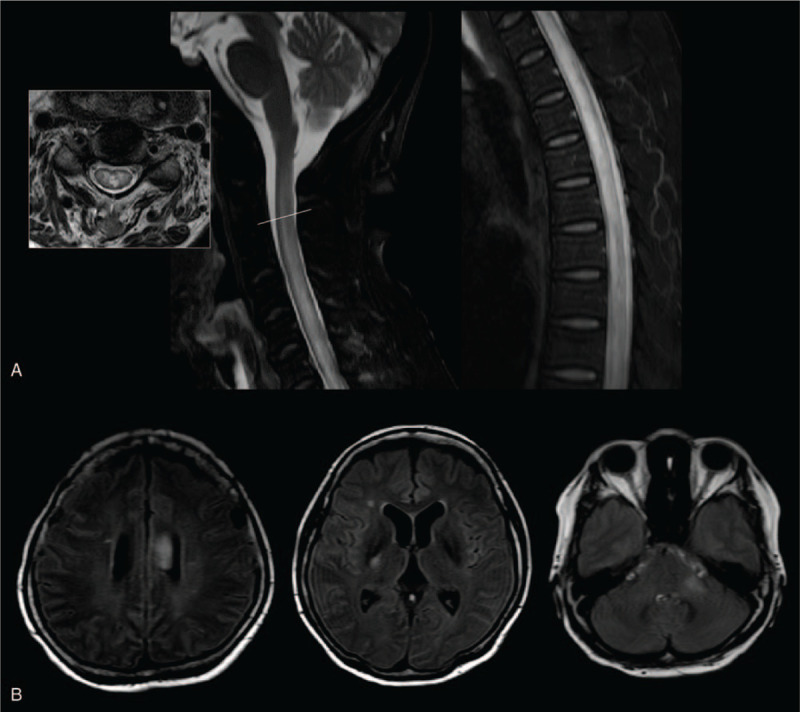
MRI of the spinal cord revealed T2 hyperintensity in whole spinal cord (A). And brain MRI showed the lesions in corpus callosum, right internal capsule and left cerebellar peduncle (B).

Magnetic resonance imaging (MRI) of the spinal cord revealed T2 hyperintensity in the whole spinal cord (Fig. 1A), and her brain MRI showed the lesions in the corpus callosum, right internal capsule, and left cerebellar peduncle (Fig. 1B). For differential diagnosis of myelitis, CSF (cerebrospinal fluid) study, autoantibodies including paraneoplastic antibody and tests for infectious causes including tuberculosis were done. Analysis of CSF revealed slight lymphocytic pleocytosis with increased protein level; CSF white blood cell counts were 7/uL, and CSF protein was 267.06 mg/dL. Serologic tests for autoimmune vasculitis, paraneoplastic antibody, serum antibodies, and CSF PCR tests for viral infection were all negative, but the AQP4 antibody was positive. Based on the international consensus diagnostic criteria for NMOSD,^[5]^ she was diagnosed as seropositive NMOSD. After the treatment with 1000 mg of intravenous methylprednisolone for 5 days, her pain was relieved, but there was no improvement of her leg weakness. Plasma exchange was not done because she and her family refused to take further treatment. One month later after hospital discharge, follow-up examination in the out-patient clinic showed no improvement; both her legs were paralyzed. The further outcome cannot be assessed because of loss to follow-up.

## 3 Discussion

The ALS and NMOSD were rare neurological diseases; recent studies reported that the prevalence of ALS and NMOSD were 3.43/100,000 and 2.56/100,000, respectively.^[6,7]^ Therefore, the coexistence of ALS and NMOSD was exceedingly rare, there have been only 2 case reports; 1 case with NMOSD preceding the onset of ALS, the other case with long-standing ALS who later developed transverse myelitis (Table 1).^[8,9]^ In this case, she was diagnosed as having ALS before the onset of NMOSD, but there was an asymptomatic AQP4 positive period for 6 years. Long-term asymptomatic AQP4 antibody positive carriers were reported infrequently, and their duration between antibody detection and symptom onset were variable which could be several years; 1 case had AQP4 antibody for 16 years without symptoms of NMOSD and the other had a 10-year asymptomatic period.^[2,3]^ According to the pathomechanism of NMOSD, AQP4 antibody is pathogenic, and it needs to access to the target antigen across the blood-brain barrier (BBB).^[1,10]^ Certain events during the asymptomatic carrier period could lead to increase the BBB permeability and initiate clinical NMOSD by the preexisting antibody; Leite et al suggested a possible role of thymus and thymectomy on the development of NMOSD,^[3]^ and the other authors reported that the mechanical opening of the BBB could be sufficient to lead to lesion formation.^[11]^

**Table 1:** Summary of reported cases with ALS and NMOSD.

	Sex	Preceding disease	Age of NMOSD onset (year-old)	Time between ALS and NMOSD (years)	Onset attack	AQP4 antibody	Remark
Falzone et al^[8]^	M	NMOSD	37	13	Myelitis	Positive	*C9orf72* mutation
Li et al^[9]^	F	ALS	54	11	Intractable hiccups and myelitis	Positive	.
This case	F	ALS	59	6	Myelitis with brain lesions	Positive	Asymptomatic AQP4 antibody carrier

There have been studies about the mechanisms of motor neuron degeneration in ALS;^[12]^ blood-brain/spinal cord barrier alterations discovered in ALS which was considered as one of the key factors for disease progression.^[4]^ And Winkler et al. demonstrated that spinal cord parenchymal accumulation of the plasma-derived immunoglobulin G with blood-spinal cord barrier breakdown in ALS.^[13]^ In our case, although the specific initiating event was not recognized, the disease of ALS could be considered as an initiating event; the disease progression of ALS along with BBB alteration could help AQP4 antibody to cross the BBB, and then facilitate the onset of NMOSD. Besides the possible BBB alteration mechanism, ALS is known to be associated with autoimmunity. One epidemiologic study showed many cases with autoimmune diseases preceding ALS including multiple sclerosis, myasthenia gravis, Sjögren syndrome, and systemic lupus erythematosus; these associations raise the possibility of shared genetic or environmental risk factors.^[14]^ And the expansion mutation of *C9orf72* in ALS could be related to the autoimmunity; a recent study suggested that *C9orf72* could regulate immune homeostasis and cause an autoimmune response in its absence.^[15]^ Therefore, *C9orf72* repeat mutation in ALS might trigger the onset of NMOSD; 1 previous case report showed the patient with NMOSD and ALS with *C9orf72* repeat mutation.^[8]^ The genetic analysis for *C9orf72* was not conducted in this patient. But *C9orf72* repeat expansion is very rare in Korea, and not the main cause of ALS in the Korean population.^[16]^ Further studies are needed to identify specific mechanisms in the concurrence of ALS and NMOSD.

ALS is a progressive motor neuron disorder, so in the advanced stage of the disease, other diseases could be undiscovered because of the profound weakness. This patient had severe weakness already but, pain, sensory loss, and bladder dysfunction could be a clue for the diagnosis of transverse myelitis which was the onset of NMOSD. Careful follow up is recommended considering the risk of developing NMOSD if patients with ALS have AQP4 antibodies.

## 4 Conclusion

This case is a rare combination of neuroinflammatory and neurodegenerative diseases, and it highlights that the consequence of ALS pathogenesis might affect the development of NMOSD.

## Author contributions

**Conceptualization:** Hye Jeong Oh, Jin Myoung Seok.

**Data curation:** Hye Jeong Oh, Jin Myoung Seok.

**Formal analysis:** Jin Myoung Seok.

**Supervision:** Yuntae Kim, Jin Myoung Seok.

**Writing – original draft:** Jin Young Kim, Jin Myoung Seok.

**Writing – review & editing:** Yuntae Kim, Jin Myoung Seok.
